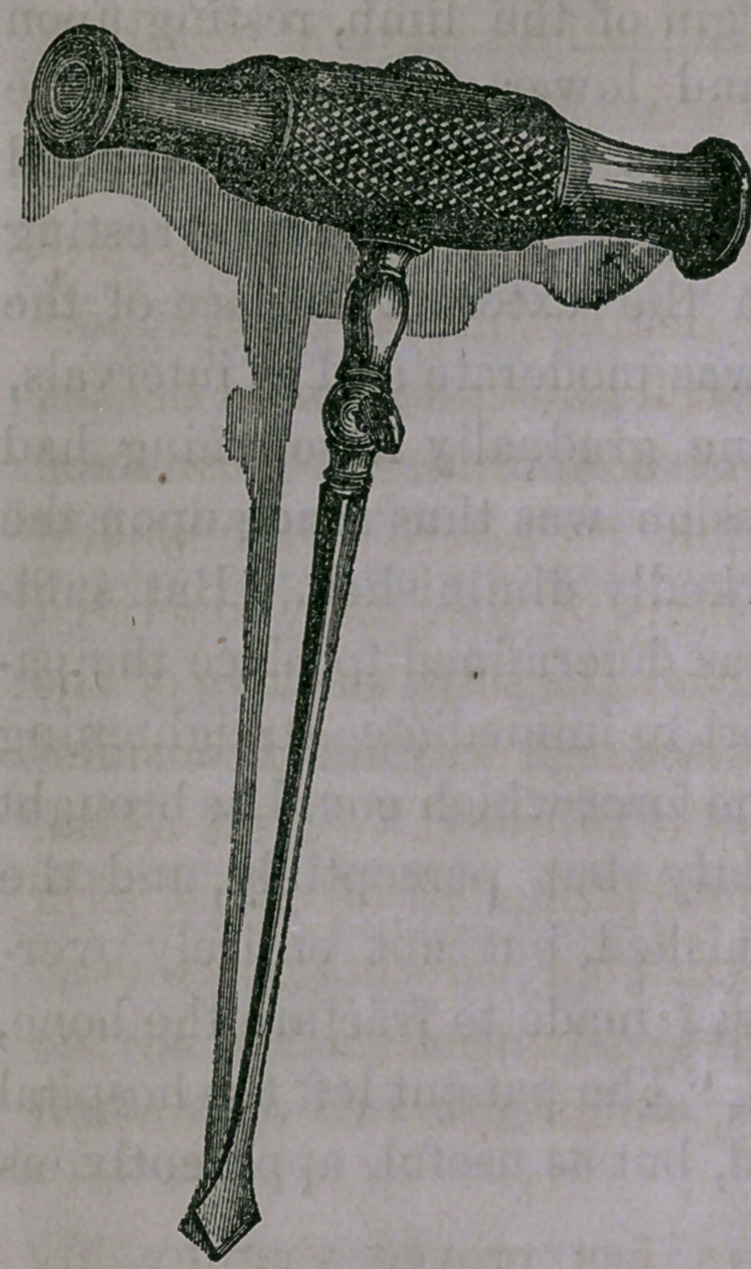# Subcutaneous Osteotomy

**Published:** 1861-05

**Authors:** Daniel Brainard

**Affiliations:** Professor of Surgery in Rush Medical College


### SUBCUTANEOUS OSTEOTOMY.

*Report of a Case of Anchylosis of the Knee Joint Successfully Treated by Subcutaneous Division of the Condyles of the Femur, with Observations on the Application of this Method of Treatment.*

BY DANIEL BRAINARD, M. D.,

*Prof. of Surgery in Rush Med. College.*

In Dec. 1853, the writer published at Paris an Essay devoted in part to the development of "a new method of treating certain deformities of the bones." The plan proposed was that of subcutaneous division, and the object of the brief notice published at that time was principally to *prendre date* and thus be able to establish a claim of priority. Experiments on the bones of living animals and certain theoretical views formed the basis of the plan as then suggested. It has since been resorted to in a number of cases differing widely from each other, and the experience thus acquired has naturally suggested some improvements in the form of instruments and the best method of using them. It is to these points that attention is now invited.

*Case.*—Wm. H. Brooks, aged 23 years, consulted me in May, 1860, on account of an anchylosis of the left knee. He had in October, 1859, received a cut on the leg below the knee, which at first gave little trouble, healing readily; but at the end of a month pain, swelling and extensive suppuration took place in and around the joint. The abscesses were opened and at the end of three months they were nearly healed.

At the time of the examination the leg was flexed to an angle of 60 degrees with the axis of the thigh, and immoveably fixed; the tibia partially dislocated outward and the patella entirely displaced upon the outside of the external condyle of the femur, where it was fixed by bony union. The lower end of the femur was considerably enlarged, numerous scars adhered to its anterior surface, and over the internal condyle was an ulcer as large as a silver dollar, which showed no signs of healing.

The patient's health was poor, and as he had previously been afflicted with eruptions on the skin, deemed scrofulous, he was advised to return home and take the iodide of potassium, which he did to the extent of five grains thrice daily for a month, during which time the ulcer on his knee healed, and his health improved so much that he was able during the summer to do every kind of hard work on a farm which the flexed position of the leg permitted.

Sept. 20, 1860, he returned in good health. The condition of the knee was the same, excepting that it was free from ulceration and tenderness and smaller than before.

The operation was performed Sept. 21, with the assistance of Dr. Edwin Powell and in presence of several medical students, as follows: Chloroform was first given. With the instrument devised for the purpose, (and hereinafter figured,) a puncture was made through the skin and tissues on the outside of the condyle, immediately above the patella. The puncture through the skin was longitudinal, but when it reached the bone the instrument was turned so as to divide it transversely and by slight movements of rotation was carried through the center of the condyles horizontally until the point could be felt beneath the skin on the inner side of the internal condyle. It was then partially withdrawn, and by passing the handle up and down and by passing it forward and drawing it backward, nearly all the cancellated structure of the bone was divided. I then directed the point to the anterior surface and cut the superficial compact layer as much as could well be done. Finding it difficult to effect this sufficiently, I withdrew the instrument from the bone, but not from the skin, and after carrying it toward the anterior surface of the femur perforated it again and repeated the attempt to divide the superficial layer in front. After having effected this as far as possible the instrument was withdrawn, and the patient lying on the bed on his back, (still insensible); I seized the ankle with my right hand, placed the left under the knee and made efforts, gradually increasing in force, to break the bone. During an effort in the direction of the flexion, and where only moderate force was being employed, a distinct snap was heard and giving way perceived of the leg. The bone had partially fractured in front, and at this point it was left, as I did not, for obvious reasons, wish to complete the fracture at once. The puncture in the skin was left open as long as any oozing of blood continued and then dressed with adhesive plaister. For some days there was a little tenderness on moving the member, but at the end of six days, Sept. 27, this was nearly gone and the puncture in the skin quite healed; there was slight swelling, but no inflammation. The member bent readily at the seat of fracture, but did not extend beyond the point where it was fixed before the operation. I now placed upon it an extending apparatus and turned the screw until a moderate amount of force was employed to straighten, when a distinct snap was heard. As the patient was not under the influence of chloroform and suffered some pain, I desisted and removed the apparatus.

Sept. 28—There was no pain except what resulted from movement and want of support. The extending apparatus was re-applied, and, after giving chloroform, the screw turned so as to straighten the leg about 20 degrees, which was done without much force.

Sept. 30—No pain; extended 15 degrees. After this time the extension was gradually increased until the member was as straight as is desirable for facility of using, which was effected Oct. 4. The bones were not displaced at the fracture, nor was pressure required to effect the object, but the seat of the old ulcerations on the knee vesicated and ulcerated at the slightest touch, so that the apparatus could be but imperfectly applied to keep up extension and preserve immobility. By great care and frequently changing the dressings this was effected, and Oct. 27, union seemed perfectly firm and the patient went about on crutches, the apparatus having been removed.

Nov. 12--He was brought before the class of Rush Medical College at the clinic. There was but a trace of the ulceration, no tenderness about the knee or the seat of fracture, which was entirely consolidated. Patient puts the member to the ground and supports himself upon it in walking.

During the whole course of treatment there has been no more inconvenience than results from a simple fracture without displacement. The result, so far as danger is concerned, is therefore as satisfactory as could be desired. In appearance the knee is perhaps less regular than after some cases of resection. I have already stated the tibia was partially dislocated outward on the femur and the patella entirely displaced before the operation. By the reduced fracture of the bone displacement of the fragments at the seat of fracture was avoided. Hence after straightening the knee projected somewhat in front, but the firmness of the limb and the readiness with which it was moved Nov. 14, seven weeks after the operation, when the patient returned home, leads me to expect that no great inconvenience will result from this irregularity. *

The case here narrated is the seventh, as far as I am informed, in which this method has been applied. As it is little known, a brief notice of each case may not be out of place here. They are given in part from the author's Report on Surgery made to the Illinois State Medical Society, 1860.

The first case in which it was resorted to, was that of a boy three years old, who had received a fracture of both bones of the leg when an infant, on whom I operated May 15, 1858.

* This patient in April, 1861, walked well and was able to do considerable work.

The deformity was so great as to render the member quite useless. The callus at the point of fracture of the tibia was perforated in two different directions, with an instrument the point of which was one-fourth of an inch in breadth. Efforts were then made to fracture the bone, but without success, and the patient was left ten days at rest, with cold water applied at times, until the inflammation, produced by the operation and efforts at fracture, had subsided. Gradual pressure was then made by means of a straight splint, with a foot-piece applied behind the leg, and a roller passed around the most projecting point of the angularity. In four weeks the member was quite straight, except a projection not considerable, resulting from the overlapping of the fragments. In eight weeks the boy was able to walk with a useful member. This case was reported in the *Chicago Medical Journal* for January, 1859.

The second case occurred in the practice of Prof. Paul. F. Eve, and was noticed in the *Nashville Medical Journal* for March, 1859.

The fracture was of six years date, of the tibia and fibula, in a boy ten years old. " Placed under chloroform and ether, with a brad-awl the tibia and fibula were several times perforated through one opening made in the skin, and the bones re-fractured by the hands. The larger bone yielded readily, but the smaller one was not sufficiently divided." "Owing to the severity of the operation and the free use of anæsthetic agents, we desisted for the time. Ten days afterwards, the fibula was again attacked by the awl, and the limb brought perfectly straight. It was now placed in a fracture box, extension and counter extension maintained as usual, and a weight—a pound of shot in a bag—retained over the point where the angle existed." I afterwards learned from Prof. Eve that his patient recovered with a straight leg.

The third case was by Prof. Pancoast, and noticed in the *Med. and Surgical Reporter* for March, 1859. It was one of anchylosis of the knee, in which extension had been tried.

The operation is thus described: "It is proposed to-day to bore holes in the bone from one external orifice in the soft parts above the knee, where the bone is least covered, and after having thus weakened the part sufficiently, to break the bone either across the knee or by apparatus." "From a single external orifice a half-a-dozen holes were bored through the bone, and after several efforts to break the bone, it was fractured, with a loud crack, distinctly audible over the whole room." The instrument employed by Prof. Pancoast was a gimlet, and he remarked that the operation was dangerous, but less so than that of cutting out a V shaped piece, which is "extremely hazardous." The case was exhibited at the following Clinic, no unfavorable symptoms having followed the operation.

Since writing the foregoing the following letter has been received from Prof. Pancoast, in reply to enquiries I had made. The result shows that the gimblet is not free from objections.

PHILADELPHIA, May 8, 1860.

*My dear Sir :—*

The result in the case of the lad, whose thigh bone I fractured after perforation, on account of anchylosis of the knee, with the leg in a flexed position, has been good. He is now running about the town, and I exhibited him to our class this winter. The foot comes well down to the ground, and the limb is almost as straight as is desirable in these cases where the knee joint is stiff.

The bone at the point of fracture forms an angle, with the apex projecting into the ham. But this projecting apex in nowise interferes with the vessels or nerves of this region. I should have had even a better result in the case, if the constitution of the lad had not been so weak and scrofulous.

After he had been taken home from the college hospital by his parents, a large abscess formed about the fracture, which was evacuated by a puncture on the side of the thigh. This was owing, I think, to the inattention and poverty of his parents; for after these causes had been provided against, the case which after the formation of the abscess had rendered me anxious, went on well. I was obliged, however, to content myself with a less complete effacement of the angularity at the knee, than would otherwise have been necessary.

The fourth case was anchylosis of the patella to the femur with false anchylosis of the knee joint, of one year's standing; the result of inflammation. The patient was a healthy girl of 16 years. As the patella could not be moved by strong efforts at flexion of the leg, while she was chloroformed, the skin of the knee was drawn forward over the patella and the broad perforator introduced from the outside and carried between the bones, which, by gentle movements of reaction, and by using it as a lever, loosened the patella with an audible snap. Adhesive plaster was applied over the puncture, and the limb left at rest for ten days, when, by moderate efforts at flexion, the patient being insensible, the patella followed the movements of the leg. Gradual extension straightened the knee in about four weeks, with little pain. This case is noticed in the Chicago Med. Journal for Feb., 1860.

The fifth occurred in the practice of Drs. English and Edgar, of Jacksonville, Ill. The case, interesting in other respects, is reported by Dr. David Prince in the May number of the Chicago Med. Journal, 1860. It was one of deformity, after oblique fracture of the upper portion of the middle third of the femur. Overlapping occured from loosening of a strip of adhesive plaster, and at the end of five weeks Dr. Prince refractured it, replaced it in dressings, which were retained seven weeks longer, and then removed the splints, union appearing to be solid and the limb straight. From want of support and proper care on the part of the patient and his parents, the callus bent so that the fragments deviated 25 or 30 degrees from a straight line, three weeks after taking off the splints. Twelve or thirteen weeks from the time of refracture, and about six weeks from the supposed time of bending, attempts to straighten it by pressure alone, were made for two days without success.

The following is Dr. Prince's description of the subsequent successful treatment:

"Several weeks now elapsed without treatment, after which three perforations were made through the region of fracture, by Brainard's drill; Dr. English, assisted by Dr. Edgar, having the treatment of the case. After waiting a week, extension was applied by means of a long splint, to the distal end of which the mechanical power of Jarvis' Adjuster had been attached. Lateral pressure was made by a sort of tourniquet with a hook passing under a splint upon the posterior part of the limb, and a screw pressing upon the convexity of the bone. It will be readily seen, that such an instrument would easily break down any bone, if the screw were forcibly turned.

The splint having been properly padded, the extension secured by adhesive straps, the counter-extension by a well cushioned perineal band fastening to the proximal end of the long splint, and the wooden ball of the screw of the tourniquet properly secured by gutta percha and padding, from pressing directly upon the prominent bony angle, extension was powerfully applied at the same time with lateral pressure, until a distinct yielding of the crooked bone was perceived. The lateral pressure was then slightly relaxed to avoid injurious pressure of the soft parts against the bone, but the extension was kept up unremittingly. In about three days the curvature disappeared, but as a precaution against accidental bending, the splint, with moderate extension, was kept on two weeks, and the thigh was afterwards protected by a starch bandage."

The sixth case occurred in the practice of Dr. Stephen Smith of the Bellevue Hospital. The patient was a boy ten years old, who had received what was decided to be "partial fracture of the tibia, with laceration of a blood vessel." The toes sloughed and when cicatrization was completed "the incurvation of the leg was considerable and efforts were made to overcome it by compression supplied at proper points, while the limb was fixed in a suitable splint." These attempts proved unavailing. Moderate efforts were then made to re-fracture the limb, but without success. Subcutaneous perforation was then resorted to, "the soft parts being opened at two points and the shaft of the tibia perforated in several directions. The external portion of the shaft, the seat of fracture and of the recently formed callus readily broke down, but the internal portion was penetrated with difficulty." The limb was kept quiet with cold water dressings for about a week, one of the openings for the perforator having suppurated when an attempt was made to straighten it with the dressings applied. To accomplish this object a strong, unyielding splint was placed upon the internal margin of the limb, resting upon pads placed upon the upper and lower extremities of the tibia; at the seat of the fracture a tourniquet was applied around the splint and limb, having its pad and screw resting directly over the fracture upon the external surface of the limb." "At first the pressure was moderate and at intervals, the object being to bend the bone gradually if softening had occurred. Considerable impression was thus made upon the limb, the deformity having markedly diminished. But sufficient was not gained." "It was determined to place the patient under chloroform and resort to immediate straightening of the limb. At applying all the force which could be brought to bear the bone yielded slightly but perceptibly, and the deformity was still further diminished, but not entirely overcome." A subsequent effort was made to fracture the bone, but it did not prove successful. "The patient left the hospital with his leg somewhat incurved, but as useful, apparently, as the other." *

If this was a case of partial fracture this fact might alone explain the difficulty of straightening. It will be seen that Dr. Smith did not think proper to follow the example of Prof. Eve and weaken the fibula. Notwithstanding that an entire absence of duties impaires the value of this case, it is still useful as showing the safety of the operation and its partial success under circumstances not the most favorable.

The only other case in which this method has been attempted, within my knowledge, came under the care of Dr. H. O. Hitchcock, of Kalamazoo, Mich. It was one of badly united fracture of the femur at the junction of the lower with the middle third. The attempt was made about six months after the accident, but it was found difficult to follow the line of callus, and as the point of one of the instruments broke in

* New York Medical Times, Nov. 3, 1860; p. 310.

the bone, the Dr. feared necrosis and cut down on the callus the next day and sawed it through.

Instruments of various forms and sizes may be required in different cases. The cut represents the one which I used over the femur and on the case of anchylosed patella of half-size. It should be made thick and so firm that in case of need it may be driven with a hammer so as to split the bone. The case of Dr. Hitchcock shows the propriety of having the instruments perfectly tempered, of the best steel, and well tried before applying them on the living subject.

*Remarks*.—The term "subcutaneous osteotomy" seems to have been first used by Prof. Langenbeck, of Berlin. From an account in Braithwaite's Retrospect, No. 31, p. 126, by Dr. P. Frank, it appears that "this brilliant acquisition to modern surgery" was first made known by Prof. Langenbeck in 1854, and the operation of sawing through the bone without extensively wounding the soft parts, first performed by him in the summer of that year.

The manner of performing it, as detailed in the paper referred to, is as follows: The integument and soft parts, including the periosteum, were divided by a strong scalpel. The bone was then bored through with a drill two lines in thickness, and a narrow saw introduced into the hole thus made, divided the greater part of the bone, the remainder being fractured. The cases which had been operated on by Prof. L. at the time of the publication were three, all of the tibia, and the result is stated to have been in all satisfactory.

It is not my intention at present to discuss the value of this operation, nor to enquire in how far it may differ from other plans of sawing bones in deep situations, several of which have at different times been proposed. It seems likely that it may be found serviceable in a certain class of cases, but what is worthy of especial notice is that this operation is not in any proper sense subcutaneous. The distinction between subcutaneous and open wounds is now well known to consist in this, that the fomer are healed without suppuration. Now Prof. Langenbeck says in his publication, "Closure of the wound by first intention happened in none of the cases operated upon, suppuration supervending from the sawn surfaces of the bone. "In the second case the discharge was not inconsiderable and a fortnight elapsed before it subsided." And again, in conclusion, "union by immediate formation of callus, as in simple fracture, cannot be expected after subcutaneous osteotomy, because the small particles of bones detached by the drill and saw act as foreign bodies, which must be eliminated by suppuration."

Experience in a number of cases has proved that by my method subcutaneous osteotomy may be performed without any suppuration; this accident having occurred only in the case of Prof. Pancoast, in which a gimlet was employed. Without going beyond facts already demonstrated, it is now certain that in cases of anchylosis where division of the bone requires to be made in the spongy structure and after fractures where the callus is not too firm, subcutaneous osteotomy by perforation may be performed with much greater advantage than subcutaneous tenotomy, comparatively speaking, since the difference in danger between open wounds of the bones (compound fractures) and those which are subcutaneous (simple fractures) is much greater than that between open and subcutaneous wounds of the soft parts.

Hand-Book for the Military Surgeon, being a compendium of the duties of the medical officer in the field, the sanitary management of the camp, the preparation of food, etc., with forms for the requisition for supplies, returns, etc.; the diagnosis and treatment of camp dysentery and all the important points in War Surgery, including gun shot wounds, amputation, wounds of the chest, abdomen, arteries, and the use of chloroform. By Chas. S. Tripler, M. D., Surgeon U. S. Army; George C. Blackman, F. R. M. S., etc. Cincinnati: Robert Clark & Co. 1861, p. 182.

The character and contents of this little volume are sufficiently indicated by the title page. The experience of Surgeon Tripler and the acquirements of Prof. Blackman, are a sufficient guarantee of the correctness of the information which it contains. It is no doubt intended to facilitate the performance of duties by those just entering on the duties of Military Surgeons, and seems well adapted to this end. At this moment we have only time to announce its appearance, but shall take occasion to return to it at some future time. The price is $1.00.

## Figures and Tables

**Figure f1:**